# Roles of Non-Coding RNA in Alzheimer’s Disease Pathophysiology

**DOI:** 10.3390/ijms241512498

**Published:** 2023-08-06

**Authors:** Edward O. Olufunmilayo, R. M. Damian Holsinger

**Affiliations:** 1 Laboratory of Molecular Neuroscience and Dementia, School of Medical Sciences, Faculty of Medicine and Health, The University of Sydney, Camperdown, NSW 2050, Australia; eddietobie@gmail.com; 2 Department of Medicine, University College Hospital, Queen Elizabeth Road, Oritamefa, Ibadan 200212, Nigeria; 3 Neuroscience, School of Medical Sciences, Faculty of Medicine and Health, The University of Sydney, Sydney, NSW 2006, Australia

**Keywords:** ncRNA, circRNA, miRNA, siRNA, piRNA, lncRNA, biomarkers, therapeutic targets

## Abstract

Alzheimer’s disease (AD) is a chronic neurodegenerative disorder that is accompanied by deficits in memory and cognitive functions. The disease is pathologically characterised by the accumulation and aggregation of an extracellular peptide referred to as amyloid-β (Aβ) in the form of amyloid plaques and the intracellular aggregation of a hyperphosphorelated protein tau in the form of neurofibrillary tangles (NFTs) that cause neuroinflammation, synaptic dysfunction, and oxidative stress. The search for pathomechanisms leading to disease onset and progression has identified many key players that include genetic, epigenetic, behavioural, and environmental factors, which lend support to the fact that this is a multi-faceted disease where failure in various systems contributes to disease onset and progression. Although the vast majority of individuals present with the sporadic (non-genetic) form of the disease, dysfunctions in numerous protein-coding and non-coding genes have been implicated in mechanisms contributing to the disease. Recent studies have provided strong evidence for the association of non-coding RNAs (ncRNAs) with AD. In this review, we highlight the current findings on changes observed in circular RNA (circRNA), microRNA (miRNA), short interfering RNA (siRNA), piwi-interacting RNA (piRNA), and long non-coding RNA (lncRNA) in AD. Variations in these ncRNAs could potentially serve as biomarkers or therapeutic targets for the diagnosis and treatment of Alzheimer’s disease. We also discuss the results of studies that have targeted these ncRNAs in cellular and animal models of AD with a view for translating these findings into therapies for Alzheimer’s disease.

## 1. Introduction

Alzheimer’s disease (AD) is a chronic degenerative condition of the central nervous system (CNS) that manifests mainly as dementia. It is the most common form of dementia and affects memory and higher executive functions, including learning, comprehension, language, and judgment, generally without effects on consciousness. The exact pathophysiological mechanisms underlying the cause of AD are still unknown. It is, however, clear that AD is a heterogeneous disease with a multifaceted etiology that includes genetic, immunologic, and environmental factors acting in concert to dysregulate homeostatic mechanisms and propagate the onset and development of the disease.

The extracellular aggregation of beta-amyloid (Aβ) peptides and the intracellular accumulation of hyperphosphorylated tau protein within the CNS are the most widely studied and recognised pathological features in AD development, while neural network and vascular theories for AD onset and development are also being actively explored [1]. Oxidative stress, mitochondrial dysfunction, autophagy, non-coding RNAs, and neuroinflammation are other processes for which new evidence of their integral roles in AD pathogenesis is constantly being discovered, further strengthening the fact that the etiological factors involved in the disease process are heterogeneous and work in concert until the final disease pathway is fully established.

Non-coding RNAs (ncRNAs) are a diverse family of non-protein-coding RNA transcripts that, due to their critical regulatory actions in multiple biological processes and disease development, are potentially useful as therapeutic targets and biomarkers for a range of physiological and pathological conditions [2]. This family of RNA molecules includes microRNAs (miRNAs), long noncoding RNAs (lncRNAs), small interfering RNAs, circular RNAs (circRNAs), and piwi-interacting RNAs (piRNAs) that interact with other RNAs, DNA, and proteins through their primary sequence and structural elements. These ncRNAs regulate biological processes including transcription, RNA turnover, translation, and post-translational assembly of proteins [3,4]. As mentioned, ncRNAs have been reported to play pivotal roles in the pathophysiologic processes that promote the onset and development of many diseases, including Alzheimer’s [5,6,7], and therefore represent potentially useful and novel therapeutic targets and disease biomarkers. In this review, we aim to highlight the current knowledge of how various ncRNAs influence pathophysiologic mechanisms and processes in AD and how these molecules may be harnessed for clinical and therapeutic benefits.

## 2. Important Pathophysiological Processes in Alzheimer’s Disease

AD is a multifactorial disease condition that involves a wide range of pathogenic mechanisms. While the most prominent risk factor for AD is age, family history, variant ε4 of the apolipoprotein E (APOE-ε4) gene, hypercholesterolemia, type-2 diabetes mellitus, and traumatic brain injury are emerging as important and sometimes modifiable risk factors for the disease [8,9,10,11]. Genome-wide association studies (GWAS) have identified several gene loci that influence the risk of AD and include the presenilin genes, *PS1* and *PS2*, *clusterin*, *complement receptor 1*, *ABCA7*, *PICALM*, *CD33*, *MS4A6A*, *MS4A4E*, *CD2AP*, *SOAT1*, and *PTGS2* [12,13]. The products of many of these gene loci are known to influence the expression of proteins involved in Aβ degradation, CNS immune regulatory processes, and cholesterol metabolism, key processes that have been identified as modulators of inflammatory and neurodegenerative components of AD pathogenesis. A number of environmental factors have also been shown to play varying roles in AD pathogenesis. However, the exact mechanisms these factors play in AD onset and progression have not been fully elucidated [14].

The accumulation of Aβ and hyperphosphorylated tau protein is a well-studied pathologic endpoint of AD and is classically identified on histologic examination as senile plaques and neurofibrillary tangles (NFTs), primarily within the hippocampus, neocortex, and other subcortical regions of the brain. Senile plaques are extracellular deposits of the Aβ peptide that are produced via cleavage of the type I transmembrane amyloid precursor protein (APP). Cleavage of the APP by α-secretase, which represents the constitutive pathway of APP processing in neurons, generates a peptide referred to as p3 and precludes the formation of toxic Aβ. Under some physiological but mostly pathological conditions, cleavage of APP by β-site APP-cleaving enzyme 1 (BACE1) activates a pathway that involves the generation of a 99 amino-acid C-terminal fragment (C99) and a soluble fragment referred to as sAPP-β. C99 is further processed by the γ-secretase complex of proteins (PS1, PEN2, Aph1, and nicastrin) to produce Aβ of lengths varying in size from 37 to 43 amino acids, with those longer than 40 amino acids being more hydrophobic and aggregating. We and others have demonstrated that levels of BACE1 protein and activity are increased in AD brain and cerebrospinal fluid (CSF) in the absence of changes in its mRNA [15,16,17,18,19]. The other protein component associated with AD pathology is the hyperphosphorylated form of the cytoskeletal protein tau, which accumulates within neurons and axons to form NFTs, with various adverse effects on neurotransmitter transport, synaptic transmission, and the regulation of apoptotic mechanisms [20]. Physiologically, tau acts to stabilise the microtubular network by binding to microtubulin, thus maintaining the integrity of the neuronal cytoskeleton and mediating the axonal transport of neurotransmitters. Increased activity of protein kinases, including glycogen synthase kinase 3β (GSK-3β), perturbation of the mitogen-activated protein kinase (MAPK) pathway, and a concurrent reduction in phosphatase activity have been shown to contribute to the hyperphosphorylation of tau and its subsequent accumulation and loss of function [21,22].

Aβ is well known to induce synaptotoxicity. Studies by Talantova and colleagues [23] revealed that the binding of Aβ to nicotinic acetylcholine receptors on astrocytes promotes the release of glutamate, which in turn activates extrasynaptic NMDA receptors (eNMDARs) on neurons, with resultant excitotoxic effects manifested by a dampening of evoked and miniature excitatory postsynaptic currents (mEPSCs) within the hippocampus [23]. The binding of Aβ to astrocytic receptors results in a cascade of cyclical events that accentuate the generation of Aβ, resulting in the generation of nitric oxide and reactive oxygen species that culminate in cellular toxicity (reviewed by [24]).

Mitochondrial dysfunction and oxidative stress act in a reciprocal, mutually potentiating manner to promote the onset and pathophysiological progression of AD [25,26]. Electron leakage during ATP (adenosine triphosphate) generation by the electron transport chain within neuronal mitochondria leads to the sequential generation of toxic reactive oxygen species (ROS) and reactive nitrogen species (RNS), all of which promote the propagation of pathologic processes involved in the disease, including Aβ generation [27,28]. Conversely, Aβ has also been shown to be a potent inducer of oxidative stress and damage within neurons [24,29].

Neuroinflammation is another important and well-established pathologic factor in Alzheimer’s disease onset and progression. Microglia are involved in the uptake and subsequent detoxification of Aβ and the mediation of various neuroinflammatory processes and pathways. During pathologic states, microglia may become overactive and undergo extensive functional and morphological changes, often resulting in the over-production of numerous pro-inflammatory cytokines and subsequent neuronal and synaptic toxicity and loss, with attendant over-production and accumulation of Aβ and tau, generating a vicious cycle of neurodegeneration [30,31]. Perturbations in cellular autophagic processes also promote the accumulation of Aβ and hyperphosphorylated tau proteins, resulting in further propagation of these toxic products [32,33].

## 3. Non-Coding RNAs: Introduction and General Regulatory Functions

The central dogma or tenet of molecular biology, as developed from studies of simpler organisms, is that DNA acts as a template for the transcription of messenger RNAs (mRNAs), which in turn serve as templates for the production of proteins via the processes of translation. Emerging research has consistently revealed an increasing number of exceptions to this rule, i.e., RNA types that do not encode for proteins, especially in more complex living organisms. These RNA molecules include the traditionally known classes of RNAs involved in translation, such as transfer RNAs (those that carry amino acids and are involved in the synthesis of proteins), ribosomal RNAs (RNAs involved in forming the machinery for protein synthesis), small nuclear RNAs, which are involved in splicing events involving mRNA transcripts, and small nucleolar RNAs that are critically involved in the chemical modification of other smaller RNAs such as ribosomal and transfer RNAs. Non-coding RNAs are broadly classified as either housekeeping or regulatory ncRNAs. Regulatory ncRNAs have been categorised based on their length into short-chain ncRNAs that include circular RNAs, short-interfering RNAs, microRNAs, and piwi-associated RNAs, and long ncRNA (lncRNA) [34,35]. As the name suggests, regulatory RNAs act as important regulators of gene expression in many essential cellular systems and biochemical interactions. Long non-coding RNA refers to species longer than 200 nucleotides in length and is known to play roles in biological processes, including epigenetic control of chromatin modification, mRNA stability, promoter-specific regulation of genes, the inactivation/lyonization of X-chromosomes, and imprinting [3].

### 3.1. Circular RNA (circRNA)

Circular RNAs possess covalently linked ends and are found in a wide variety of organisms, ranging from non-cellular pathogens such as hepatitis delta virus [36] to various eukaryotic organisms, where they are typically produced from errors in mRNA splicing [37,38]. They originate from a process termed back-splicing and may contain segments of introns, exons, or even non-coding intergenic regions [39]. Since eukaryotic DNA contain split genes (segments of exons separated by noncoding sequences), precursor mRNAs (pre-mRNAs) must undergo modifications that ensure non-coding mRNA segments are removed and protein-coding exons are combined in the process of splicing. An error in the splicing mechanism can lead to the joining of the two ends of a single exon or a preceding exon, leading to the formation of circRNA [40,41]. The exact mechanisms by which the splicing machinery selects particular RNA sequences or segments to circularize are not fully understood, but the presence of inverted repeats in neighbouring introns and/or exons may be responsible [38,40]. Linear mRNAs themselves may also circularize during translation via interactions between factors that combine the 5′ and 3′ ends of the mRNA, via processes such as direct covalent interactions, protein bridging, or even via Watson-Crick base pairing [42]. Evidence suggests that circRNAs are resistant to most RNA degrading machines, which act by initially binding to the free ends of linear RNAs and, therefore, large amounts of circRNA may accumulate within the cytoplasm of cells [43].

A circRNA that is predominantly found in human and mouse brains is ciRS-7. Located on chromosome Xq27.1, ciRS-7 consists of approximately 1500 nucleotides and harbours over 70 highly conserved microRNA binding sites. MicroRNAs avidly bind to transcripts with complementary sequences, and this particular circular RNA may bind up to 20,000 miR-7 microRNAs per cell [41], in an interaction described as “sponging” [44], with resultant microRNA degradation. This suggests that circRNAs may play pivotal roles in regulating the number and activity of microRNAs. Beyond their actions in the regulation of microRNAs, circRNAs may also sequester other forms of RNA (in addition to microRNA) as well as RNA-binding proteins, forming large RNA-protein complexes [42].

### 3.2. MicroRNA (miRNA)

MicroRNAs typically consist of 21–23 nucleotides and exert gene-regulatory functions by forming sequence-specific base pairs with mRNAs to repress translation and/or initiate mRNA degradation [45]. Lee and colleagues [46] discovered that *lin-4*, a gene that controls larval development in *C. elegans*, coded for a pair of small RNAs. The first RNA was found to be 22 nucleotides in length, and the second, which contained 61 nucleotides, was proposed to be a precursor of the shorter form as it folded into a loop and underwent further processing [46]. The shorter lin-4 RNA is now widely considered the first member of the large class of small regulatory RNAs referred to as microRNAs [47,48,49]. The production of miRNAs commences with the synthesis of primary miRNA transcripts (pri-miRNAs) by RNA polymerase II. Pri-miRNAs possess one or more stem loop structures that are subsequently processed by the Microprocessor complex in the nucleus, consisting of a ribonuclease III enzyme, Drosha, and the RNA-binding protein DiGeorge syndrome critical region 8 (DGCR8) [50]. The small hairpin precursor miRNAs (pre-miRNAs) that are released characteristically have a 2-nuleotide 3′ overhang. These pre-miRNAs are transported through nuclear pores into the cytoplasm bound to Exportin5/RanGTP and, when released, are further processed by the RNase III endonuclease, Dicer, into a miRNA duplex, which is then loaded onto the Argonaute (AGO) protein family (AGO1-4 in humans) in an ATP-dependent manner [51]. There are currently approximately 1915 identified microRNA precursors in the human genome, of which 725 are high-confidence identifications, with these precursors producing active microRNAs with one or both strands [52,53].

MicroRNAs exert their effects by participating in ribonucleoprotein (RNP) complexes, referred to as either miRNA-induced silencing complexes (miRISCs) or miRNPs, with the AGO family of proteins being the most important and best characterised members of the complex. The microRNAs bind to complementary sites on the target mRNAs and, together with the components of the miRISC, guide members of the Argonaute protein family to either degrade the bound mRNA or inhibit the initiation of its translation into protein products [54,55]. mRNA degradation is specified by microRNA if there is sufficient complementarity between the two, while repression of translation is specified if the complementarity is inadequate to stimulate degradation [56,57]. When degradation is favoured by the microRNA, the excision is made precisely between the mRNA nucleotides paired to the 10th and 11th residues of the microRNA [58,59]. Repression of mRNA translation may be achieved by microRNAs either via the inhibition of the actions of ribosomes and their components on the target mRNA or by causing an essentially non-productive translation process via the degradation of nascent polypeptides being produced from the ribosomal complexes [60]. Via their influence on a vast number of different mRNAs, microRNAs have the ability to modulate the expression of genes that participate in the regulation of a wide variety of cellular metabolic or developmental processes and pathways and possibly even act as switches for the expression of some of these genes.

### 3.3. Small Interfering RNA (siRNA)

Consisting of approximately 21 nucleotide-long double-stranded RNA molecules, small interfering RNAs are known to exert important gene regulatory effects via the process of RNA interference (RNAi). RNAi refers to the biological processes involved in the silencing of genes via the degradation of complementary mRNAs via the actions of double-stranded RNAs (dsRNA) [61]. RNA interference has been extensively studied for its potential benefits in the study of genomics and gene functions and in the treatment of various classes of diseases, including cancer and neurodegenerative diseases [62,63]. RNA interference as a phenomenon was first discovered in plants and was then demonstrated in *C. elegans* [64].

The production of siRNAs involves the cleavage and processing of longer double-stranded RNAs (dsRNA) into siRNAs. These siRNAs are generally characterised by a double nucleotide overhang at the 3′ end of each RNA strand, generated via the actions of Dicer [65,66], the endoribonuclease also involved in miRNA synthesis. When formed, siRNAs, similar to miRNAs, are incorporated into RNA-induced silencing complexes (RISC) [67,68,69]. Following binding to the RISC complex, the siRNA strands separate, and the strand with the most stable 5′-end integrates into the RISC complex. The single-stranded antisense RNA aligns the now functional RISC complex with the target mRNA and, employing the catalytic RISC protein, degrades the target [70,71].

One may note similarities between the actions of miRNAs and siRNAs and question whether the similarities in their primary actions translate to similarities in their effects on protein expression and ultimately cellular function and survival. Okamura and Lai [72] argue that the observations that worms and flies with dysfunctional RNA interference pathways are generally normal and fertile, while those with dysfunctional miRNA pathways often suffer lethal consequences, suggest that the role of endogenous siRNAs (and essentially, RNA interference) fundamentally differs from that of miRNA regulation. Additionally, animal studies show that many miRNAs are highly evolutionarily conserved [73,74], while most pseudogenes that generate siRNAs are poorly conserved [75]. More investigations are needed to clarify the differences between gene regulatory processes by siRNAs and miRNAs in mammals and humans.

Due to their ability to silence many disease-causing genes, siRNAs have gained attention as potential therapeutic agents. Considerable efforts have been expended in the development of siRNA-based therapies for a wide range of metabolic disorders, cancers, and neurodegenerative diseases, as well as in in vivo and in vitro studies of single gene functions [76,77].

**Figure 1:**
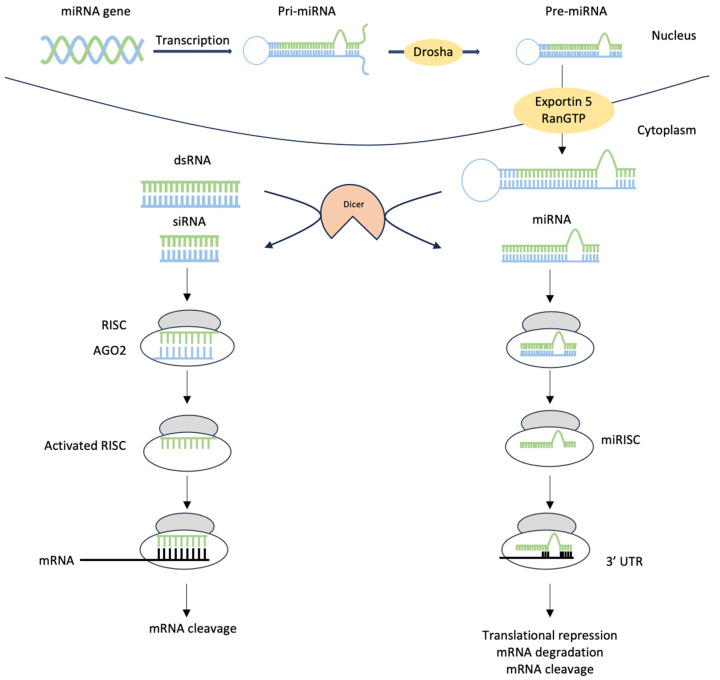
Common mechanisms of gene silencing driven by siRNA and miRNA. siRNA: dsRNA, produced from an miRNA gene is transcribed into a primary miRNA (pri-miRNA) and then processed by Drosha into precursor miRNAs (pre-miRNAs) in the nucleus. Pre-miRNAs are exported out of the nucleus into the cytoplasm, where it is processed by Dicer into siRNA, which is loaded into the RNA-induced silencing complex (RISC). Argonaute (AGO2), which is a component of RISC, cleaves the passenger strand of siRNA that is then degraded by cellular machinery. The guide strand then guides the active RISC to the target mRNA. The complementary binding between the guide strand of siRNA and the target mRNA leads to the cleavage of mRNA. miRNA: Transcription of an miRNA gene is performed by RNA polymerase II in the nucleus to generate pri-miRNA, which is then cleaved by Drosha to form pre-miRNA. The pre-miRNA is transported by exportin 5 to the cytoplasm, where it is processed by Dicer into miRNA. Similar to siRNA, the miRNA is loaded into the RISC, where the passenger strand is degraded. The miRISC is guided by the remaining guide strand to the target mRNA through partial complementary binding. Unlike siRNAs, miRNAs are known to post-transcriptionally regulate target mRNAs through the 3′-UTR, which interacts mainly with the 5′-end of the target mRNA. Image adapted from [78].

The mechanisms leading to gene silencing driven by siRNAs and miRNAs are outlined in Figure 1.

### 3.4. Piwi-Interacting RNAs (piRNAs)

P-Element-induced wimpy testis (PIWI)-interacting RNAs (piRNAs) are a class of non-coding RNAs that interact with and guide PIWI-clade Argonaute proteins to silence transposable elements in genes and regulate gene expression, particularly in germ cells. PIWI proteins were initially discovered in *Drosophila melanogaster* germline cells, where they were found to be important in germline maintenance and renewal [79,80]. Subsequent studies have demonstrated that piRNAs are also expressed in a tissue-specific manner within many human somatic tissue types, where they play regulatory roles in transposon silencing, gene regulation, epigenetic regulation, genome rearrangement, germ stem cell maintenance, and spermatogenesis [81].

Three groups of piRNAs have been identified: Transposon-derived piRNAs are the best-studied piRNAs and are transcribed from both genomic strands, yielding sense and antisense piRNAs; piRNAs are typically produced from 3′ untranslated regions (UTRs) of mRNAs, whereas lncRNA-derived piRNAs are produced from the entire transcript [82,83]. Unlike stem-loop or double-stranded miRNA and siRNA precursors that are processed by the RNAse III-like enzyme, Dicer, piRNAs are transcribed as large, single-stranded precursors, which subsequently undergo post-translational processing independently from Dicer [81,84,85]. This process occurs on or near the mitochondria and is largely controlled by the endonuclease ZUCCHINI/PLD6/MITOPLD [86]. PiRNA precursors are devoid of secondary stem-loop/hairpin structures, unlike those identified in miRNA. Like most other ncRNAs, the piRNA precursors require post-transcriptional processing to attain full structural maturity and function effectively.

As discovered in germline cells, piRNAs interact with members of the Piwi subfamily of proteins, including the Argonaute proteins Aub, Piwi, and AGO3, in order to perform their numerous functions [87,88]. Once associated with Piwi proteins, piRNAs undergo further posttranscriptional modifications whereby the 3′-end of the piRNA is 2′-O-methylated, protecting the piRNA from degradation. The Piwi-piRNA complex forms RNA-induced silencing complexes (RISC) and guides the silencing complexes to RNA and transposon targets [89]. Transposons (or transposable elements) are genetic elements that possess the ability to constantly change their location within the genome via numerous mechanisms. They were found to often promote deleterious effects in many living organisms, including humans, classically with their discovery in the plasmids of antibiotic-resistant bacteria and more recently in altered gene regulation seen in many genetic diseases and cancers [90,91]. PiRNA-Piwi complexes mediate RNA interference and transposon silencing by facilitating a state of chromatin repression and heterochromatin formation [92]. The silencing pathway commences when piRNA-Piwi complexes recruit the epigenetic factor HP1a (heterocromatin protein 1a), which in turn recruits Su(var)3-9, a histone methyltransferase [93,94]. Su(var)3-9 catalyzes the trimethylation of the DNA packaging protein histone H3 at lysine 9 (H3K9me3). The gene regulatory functions of piRNAs in a wide range of contexts, including signal transduction and tumour suppressor pathways, have been described [95,96]. The potential efficacy of piRNAs and Piwi proteins as diagnostic/prognostic markers and novel therapeutic agents has also been extensively described [97,98,99,100,101].

### 3.5. Long Non-Coding RNA (lncRNA)

Long non-coding RNAs are ncRNAs that are longer than 200 nucleotides. They constitute a large and heterogeneous group of RNAs with varying genomic origins, biogenesis, and functions. Estimates place the number of lncRNA genes within the human genome at ~16,000 [102,103]. Based on their cellular localization and interactions with other biochemical components within cells, lncRNAs are known to perform various regulatory and modulatory functions for chromatin, cytoplasmic mRNAs, membraneless nuclear bodies, and signalling pathways. Many of these functions ultimately influence the pathophysiological processes in numerous neuronal disorders, including immune, cancer, and neurodegenerative conditions, with some sequencing studies revealing considerable amounts of dysregulated lncRNAs in the human neocortex [104].

Similar to mRNAs, most lncRNA species are transcribed by RNA polymerase II and are post-transcriptionally processed to include 5′-m7G (methylguanosine) capping and 3′-polyadenylation [105]. In contrast to mRNAs, a large proportion of lncRNAs are sequestered within the nucleus [106,107]. This striking difference in cellular localization may be explained by the fact that lncRNA genes are generally less evolutionarily conserved, contain fewer exons, and are expressed in significantly lower amounts compared to mRNAs [108,109]. The relatively lower rates of transcription and expression of lncRNA genes have been proposed to be linked to the repressive modifications on histone proteins at the loci of gene promoters [110,111]. In addition, the actions of RNAPII with dysregulated carboxyl terminus phosphorylation result in weakly spliced lncRNA that do not respond to polyadenylation-induced transcription termination, leading to the accumulation of lncRNAs on chromatin with subsequent RNA exosome-mediated degradation [112]. These findings attempt to explain the largely nuclear localization of lncRNAs and imply that these mechanisms would need to be negotiated in order for lncRNAs to transit into the cytoplasm and perform some of their functions. The cytosolic translocation of long and A/U-rich RNA transcripts (such as lncRNAs) has been found to be dependent on the exportin NXF1 [113], after which they are either distributed to the cytoplasm in association with RNA-binding proteins or sorted to specific organelles [114].

lncRNAs, via mechanisms that involve DNA and histone modifications, are known to influence the selective repression or activation of various genes [115]. They have been shown to promote gene expression via the recruitment of histone H3K4 methyltransferases [98,116] and repress gene expression by binding and activating DNA methyltransferases such as DNMT1 and DNMT3b [117,118]. lncRNAs also exert influence on proteins, enzymes, and their interactions, with important effects on posttranslational modifications and fundamental cellular signalling pathways. The NF-kappaB (NF-κB)-interacting lncRNA (NKILA) has been shown to bind NF-κB/IκB in a ternary complex, obscuring its phosphorylation site and essentially inactivating the NF-κB signalling pathway [119]. Moreover, lncRNAs within dendritic cells have been shown to regulate protein expression and modifications via their interactions with the transcription factor signal transducer and activator of transcription 3 (STAT3) and the tyrosine phosphatase SHIP1 [120].

These studies suggest the feasibility of employing lncRNAs as potential biomarkers and therapeutic targets for a wide range of diseases.

## 4. Roles of Non-Coding RNAs in Alzheimer’s Disease Pathogenesis

A growing body of literature consistently implicates noncoding RNAs (ncRNAs), in particular miRNAs and lncRNAs, in AD pathogenesis. These ncRNAs have been shown to contribute via numerous pathways to amyloid-β (Aβ) peptide and tau accumulation, neuroinflammation, neuronal loss, and other known pathomechanisms by which AD states become established.

### 4.1. Roles of MiRNAs in Alzheimer’s Disease Pathophysiology

MiRNAs are expressed in large numbers within the CNS and display region- and age-specific expression patterns [121,122], with a specific subset shown to be expressed in the hippocampus and cortex in adult mice [123]. MiRNA expression profiles also depend on the subtypes of neurons (e.g., glutamatergic vs. GABAergic neurons) as well as their locations within the cell (e.g., distal axons vs. synaptic fraction). Some miRNAs known to populate synaptic areas include miR-7-5p, miR-29a-3p, miR-137-5p, miR-200c-3p, miR-318-3p, miR-322-5p, and miR-339-5p [124], while those that populate distal axons include miR-16-5p, miR-204-5p, and miR-221-3p [125]. These co-localization patterns suggest that different miRNAs may play distinctive functions in diverse regions of the nervous system.

Studies into the roles of miRNAs in CNS function have revealed that these small, 21–23 nucleotide sequences have been shown to be involved in important processes including neurogenesis, neuronal plasticity, synaptic function, memory, and learning [121,122]. The miRNAs miR-106a-5p/363-3p, miR-17-5p/92-3p cluster, and miR-106b-5p/25-3p have been shown to be important during brain development [126,127], while miR-124-3p and miR-9-5p are critical for neurogenesis, axonal development, and neuronal migration [128,129,130]. MicroRNAs also exert influences on cells within the CNS other than neurons. MiR-125-5p and Let-7b-5p regulate astrocyte differentiation [131], while miR-338-5p and miR-138-5p are involved in oligodendrocyte differentiation [132]. Studies in mice have shown that the deletion of the gene for Dicer, which cleaves miRNA precursors, enhanced behavioural performance, and post-tetanic potentiation (PTP), which reflect synaptic plasticity, with altered levels of the synaptic proteins BDNF and PSD95 and altered the morphology of dendritic spines [133]. These findings imply that miRNAs play crucial roles in memory and learning processes, with miR-134-5p specifically shown to affect long-term memory [134]. Attempts have also been made to describe the roles of miRNAs in neuroinflammation. MiR-146a-5p negatively regulates inflammation by inhibiting the toll-like receptor 4 (TLR4) signalling pathway [135]. Additionally, pro-inflammatory microglia have been shown to express increased miR-155-5p and decreased miR-146a-5p levels, changes that are required for the transition to a pro-inflammatory state [136].

Many studies have shown significant dysregulation of miRNAs in the CNS and tissues of AD animal models and human subjects.

#### 4.1.1. Roles of MiRNAs in Aβ Production and Clearance

Amyloid plaque deposition in the brain is a pathological hallmark of AD, and abnormalities in Aβ production and metabolism are known to contribute to the disease. Aβ is produced by the sequential proteolytic cleavage of the amyloid precursor protein (APP) by enzymes known as BACE1 (β-site APP cleaving enzyme 1) and γ-secretase (a complex of four proteins with presenilin 1 (PS1) acting as the key proteolytic enzyme within the complex). Following cleavage of APP by β-secretase, the membrane-tethered APP C-terminal fragment is then processed by γ-secretase to generate Aβ and the APP-intracellular domain (AICD), which can translocate to the nucleus and act as a potential transcription factor. We and others have reported that β-secretase protein and activity levels are increased in AD brain and cerebrospinal fluid in AD subjects [15,18,19] and that BACE1 represents the rate-limiting step in Aβ production [15]. Mutations in the APP and PS1 genes have been associated with an early-onset form of AD that results in the production of longer and more fibrillogenic forms of Aβ. Drugs targeting BACE1 and PS1 have entered various stages of clinical trials, but due to the roles played by these enzymes in other key proteolytic pathways, many of these drugs have failed to progress to the clinic.

MiRNAs have been shown to be involved in Aβ pathology via their influences on APP and the enzymes that produce Aβ. The expression of miR-16, miR-29a/b-1 and c, miR-186, and miR-195 has been shown to be decreased in the brains of patients with AD and AD mice [5,137,138,139,140]. MiR-16 has been shown to regulate the expression of both APP and BACE1 as well as nicastrin, a component of the γ-secretase complex of proteins [141]. In addition, miR-16 was also shown to decrease levels of total tau phosphorylation in neuronal cell lines [141]. Translating their findings in vivo, Parsi and colleagues [141] discovered that miR-16 was capable of decreasing endogenous BACE1 and tau protein levels in a dose-dependent manner in wildtype mouse brains. Herbert and colleagues [5] reported that miR-29a and -29/b-1 were significantly decreased in a cluster of AD subjects who had an elevated level of BACE1 in the brain. We and others have shown that BACE1 protein levels are significantly elevated in the brain and cerebrospinal fluid of AD subjects [15,16,17,18,19] in the absence of changes in BACE1 mRNA expression [15]. BACE1 is the rate-limiting enzyme in Aβ generation, as we showed a more than 2-fold accumulation of β-CTF in the AD brain of subjects with elevated BACE1 expression [15]. The observation by Herbert and colleagues provides a potential explanation for the increase in BACE1 protein in the absence of mRNA changes. MiR-195 is another modulatory non-coding RNA sequence that is altered in the AD brain. Cao and colleagues [142] discovered that miR-195 was reduced in the parietal cortex of MCI and early AD subjects carrying a single ApoE ε4 allele compared to ApoE ε4^−/−^ subjects. They also observed a significant reduction in miR-195 expression in female compared to male subjects. Interestingly, this group also discovered that levels of miR-195 were decreased in 12-month-old ApoE4^+/+^ mouse brains compared to ApoE3^+/+^ mice, recapitulating results observed in humans. Intriguingly, overexpression of miR-195 rescued cognitive deficits and significantly reduced amyloid burden in the ApoE4 mouse models [142], highlighting the therapeutic potential of miR-195. This non-coding RNA species is known to regulate phosphoinositol biphosphate (PIP_2_) and synaptojanin 1 (synj1) levels in the brain. Synj1 is a brain PIP_2_-degrading enzyme, and overexpression of miR-195 reduces expression levels of synj1 [142].

A major mechanism of miRNA function is its binding to the 3′ untranslated regions (UTR) of target genes. Zhang and colleagues [143] showed that overexpression of miR-188-3p suppresses BACE1 gene transcription and expression in 5xFAD transgenic (Tg) mice. This was shown to reduce Aβ production and its neurotoxic and synaptotoxic effects [143], with the opposite effects occurring when a miRNA sponge that decreases miRNA expression was employed. MicroRNAs may also display effects on Aβ production via their direct effects on genes other than BACE1. Peroxisome-proliferator-activated receptor gamma (PPARγ) is known to inhibit the expression of the BACE1 protein, suppressing Aβ production and accumulation [144]. Liu et al. [145] showed that miR-128 in the cerebral cortex of 3xTg AD mice targets and inhibits the expression of PPARγ, suggesting that knockout of miR-128 may reduce amyloid plaque formation and deposition, in contrast to other highlighted micro-RNA studies. A number of miRNAs have also been proven to reduce the generation of amyloid plaques via their inhibitory effects on APP. MiR-346, miR-101, miR-101a-3p, and miR-384 are known to inhibit APP production (and consequently, amyloid plaque generation) via their actions on either the 3′ or 5′ UTRs of the APP gene, and these microRNAs have been noted to be downregulated in animal models and AD subjects [146,147,148].

The epsilon 4 allele of apolipoprotein E (APOE ε4) is a major risk factor for AD, especially the late-onset form of the disease. Apolipoproteins are known to be involved in lipid metabolism and the exact mechanisms by which they contribute to the disease process are still under investigation [149]. The clearance of Aβ within the CNS is affected by the actions of microRNAs on APOE mRNAs. MiR-1908 interacts directly with the 3′-UTR of ApoE mRNA and subsequently reduces ApoE mRNA and APOE levels, with a resultant diminution of ApoE-mediated Aβ clearance observed in AD patients [150]. ATP-binding cassette transporter A1 (ABCA1) is an important regulator of APOE lipidation and Aβ clearance. Kim et al. [151] showed that miR-33 in the brains of AD mice suppresses ABCA1 expression via its interaction with the 3′-UTR of the ABCA1 mRNA in neural cells. Thus, miR-33 overexpression significantly increases extracellular Aβ accumulation by impairing neuronal Aβ clearance, underscoring its potential as a target for the treatment of AD [151].

#### 4.1.2. Roles of MiRNAs in Tau Expression and Phosphorylation

The microtubule-associated protein tau, generally localized within axons, is responsible for the stabilization of microtubular components and axonal transport. The abnormal hyperphosphorylation and aggregation of tau result in the formation of neurofibrillary tangles (NFTs), a common pathological hallmark of AD [152].

Recent studies have revealed that miRNAs influence the regulation of tau protein. Investigations on transgenic mice by Hernandez-Rapp and colleagues [6] revealed that miR-132 inhibits the expression of tau protein, with its deficiency leading to increased expression, phosphorylation, and aggregation of tau, leading to deleterious effects on long-term memory. Similarly, Santa-Maria and colleagues [153] showed that miR-219 alters the expression of tau mRNA via its interaction with its 3′-UTR. Contrasting effects have, however, been reported. Increased expression of miR-146a has been shown to enhance abnormal tau hyperphosphorylation in the brains of AD subjects [154]. Rho-associated coiled-coil-containing protein kinase 1 (ROCK1) dephosphorylates tau by phosphorylating and activating the Phosphatase and Tensin Homolog (PTEN) protein. ROCK1 mRNA is a target of miR-146a, and thus, overexpression of this miRNA will indirectly result in the accumulation of hyperphosphorylated tau, resulting in memory impairments, as shown in animal studies [154,155].

Other indirect relationships have also been described. MiR-512 has been shown to inhibit the expression of the genes for the anti-apoptotic proteins MCL1 and cFLIP, and a reduction in the levels of this microRNA has been associated with the hyperphosphorylation of tau in AD brains [156]. MiR-137 has been shown to inhibit tau hyperphosphorylation via its actions with the 3′-UTR of the calcium voltage-gated channel subunit alpha-1 C (CACNA1C) mRNA, suppressing expression of its protein product in the hippocampus and cortex of AD mice, which is presumably involved in tau hyperphosphorylation [157].

#### 4.1.3. Roles of MiRNAs in Neuronal Proliferation and Loss

MicroRNAs exert influences on neuronal apoptosis via their regulatory actions on target genes and signalling pathways involved in the process. Brain-derived neurotrophic factor (BDNF), a member of a family of neurotrophic factors, promotes neuronal survival, protects against neuronal apoptosis, mitochondrial dysfunction, and oxidative stress, aids the formation of new synapses, and enhances neuronal plasticity while offering protective effects against Aβ toxicity [158,159]. BDNF levels are known to be reduced in Alzheimer’s disease [160]. The microRNA miR-206, found to be upregulated in the serum of AD patients [161], has been shown to decrease BDNF expression by binding to the 3′-UTR of BDNF mRNA [162]. Decreased levels of BDNF will potentially leave neurons vulnerable by exposing them to various neurotoxic consequences without adequate trophic support.

Epidermal growth factor receptor (EGFR), acting via MAPK/ERK and PI3K/AKT signalling pathways, is known to play crucial roles in neuronal plasticity, survival, and protection from neurotoxicity [163]. Yang and colleagues [164] reported that levels of miR-133b were substantially lower in AD patients compared to healthy controls and that miR-133b levels were positively correlated with scores on the mini-mental state examination (MMSE) in the study population. They also showed that miR-133b significantly attenuated Aβ-induced neuronal apoptosis, underscoring its neuroprotective actions and potential as a biomarker [164].

Studies by Wang and coworkers [165] have demonstrated that miR-222, via its interactions with the protein p27*^Kip1^*, plays important regulatory roles in cell cycle progression and neuronal proliferation and may contribute to the pathological processes in AD. P27*^Kip1^* inhibits the phosphorylation of the retinoblastoma (Rb) protein, a pivotal cell cycle checkpoint protein. Phosphorylation of Rb is required to progress the cell from the G1 phase of the cell cycle to the synthesis (S) phase. Increased levels of p27*^Kip1^* would essentially halt cell proliferation in the G1 phase. A recent study revealed significant decreases in the level of miR-222 in the serum of mild and moderate AD patients [166]. The finding of decreased miR-222 early in the disease highlights the potential of this miRNA as a biomarker for AD and should be further investigated.

#### 4.1.4. Roles of MiRNA in Neuroinflammation

Neuroinflammation plays a pivotal role in the pathophysiological processes involved in AD, with many complex and often reciprocal interactions described between inflammatory mediators, cells, and the pathological hallmarks of the disease. The accumulation of Aβ results in cellular reactions, including the chronic activation of microglia, which, during the process of clearing Aβ, release inflammatory cytokines and chemokines that propagate neuroinflammation [167]. MicroRNAs exert regulatory effects on the intracellular pathways and functions of numerous mediators of neuroinflammation.

Liu and colleagues [168] reported that increased expression of miR-155 in the hippocampus of AD rats correlated with elevated expression and functioning of pro-inflammatory cytokines. Knockout of miR-155 significantly suppressed caspase-3 levels and other inflammatory signalling pathways that it mediated, with corresponding improvements in learning and memory in the rats [168]. Studies by Guedes and colleagues [169] also revealed similar findings, with miR-155 expression noted to enhance the production and actions of the inflammatory mediators IL-6 and interferon beta (IFNβ) in activated astrocytes and microglia. This effect is associated with the direct inhibitory effects of miR-155 on the expression of SOCS-1 (suppressor of cytokine signalling 1), a potent inhibitor of inflammatory mechanisms, as observed in 3xTg AD mice used in this study, suggesting that miR-155 may be a potent neuroinflammation-based therapeutic target for AD [169].

The microRNAs miR-132 and miR-212 have also been associated with neuroinflammation in AD. Hadar et al. [170] demonstrated that the expression of these two miRNAs was upregulated in lymphoblastoid cells (LCLs) from AD patients, with a corresponding decrease in the expression of silent information regulator 1 (sirtuin1, SIRT1), a known target of miR-132 and -212. SIRT1 is known to play critical anti-inflammatory roles in various disease states typified by inflammation, including AD, where it exerts neuroprotective effects via specific mechanisms [171], and the downregulation of the transcription of its gene by these microRNAs may serve to propagate neuroinflammatory processes in AD.

#### 4.1.5. Roles of MicroRNAs in Oxidative Stress

Oxidative stress plays an important role in the pathogenesis of AD [28,172]. It is closely associated with mitochondrial dysfunction and is characterised by an imbalance between the generation and degradation of reactive oxygen and reactive nitrogen species (ROS and RNS) [28,173]. There are complex and often reciprocal relationships between ROS/RNS levels and various pathological factors in AD. Elevated levels of ROS may initiate the processing of the amyloid precursor protein, promote Aβ accumulation, and activate various signalling pathways that propagate the development and progression of AD states [28,29].

Notch signalling is known to be important for differentiation, proliferation, regulation of apoptosis, and mediation of oxidative stress in a number of cell types, including neurons, especially during the course of AD [174,175]. Chen et al. [176] demonstrated that Hairy and enhancer of split (Hes)-related with YRPW motif protein 2 (HEY2), an important transcription factor associated with Notch signaling, is a target of miR-98 and that inhibition of HEY2 by this microRNA resulted in inactivation of the Notch signalling pathway in AD mice. The inactivation led to the suppression of Aβ production and reduced mitochondrial dysfunction and oxidative stress in the mice [176].

In mice harbouring both the APP Swedish and PSEN1 delta 9 mutations (APPswe/PSEN1Δ9), Wang and colleagues [177] discovered abnormal expression of the oxidative stress-associated microRNAs miR-34a, miR-34c, and miR-98 between 3 and 6 months of age. Intriguingly, this time period corresponds with the deposition of Aβ in the hippocampus in this model [178]. Other studies have described similar effects of oxidative stress on microRNAs in AD states. Reactive oxygen species have been shown to upregulate miR-20a levels in primary hippocampal neurons [179], and this microRNA is known to reduce Aβ formation and accumulation by its inhibitory effects on APP mRNA transcription [180], indicating a protective role for the miRNA.

Table 1 provides a summary of some dysregulated miRNAs found in AD tissues.

### 4.2. Roles of Small Interfering RNAs in Alzheimer’s Disease Pathophysiology

Numerous studies have investigated the role of siRNAs in AD pathophysiology. Hérard and colleagues [181] were the first to demonstrate siRNA-mediated, in vivo repression of a protein at the synapse. They showed that intraocular-injected siRNA was transported into retinal ganglion cells and subsequently drastically reduced the volume of newly synthesised and axonally transported APP and amyloid precursor-like protein 2 (APLP2) in retinal termini in the adult rat brain [181]. These findings suggest that alterations in the levels of APP/APLP2 and their turnover at the synaptic termini could potentially perturb synaptic function and possibly facilitate pathogenic processes in AD.

Studies by Miller and colleagues [182] showed that siRNAs siT10/C11, synthesised to target wildtype APP and APPsw expressed in COS-7, caused highly specific silencing of their targets, as demonstrated by both immunofluorescence and western blot analyses. They also demonstrated significant suppression of tau expression by synthesised siRNAs targeting wildtype and mutant tau (V337M) expressed in COS-7 cells [182].

The microtubule affinity-regulating kinase 2 (MARK2) protein has been shown to be an important generator of tau as it phosphorylates serine 262 of the tau protein [183]. Azorsa and colleagues [184] showed that siRNAs targeting MARK2 reduced its expression by as much as 95%, with a resultant 26% reduction in 12E8 phosphorylated tau but a less significant change in total tau protein levels [184]. In addition, Azorsa and colleagues also demonstrated that siRNA-mediated knockdown of EIF2AK2 (eukaryotic translation initiation factor 2 α kinase 2) resulted in a significant reduction in pS262 tau levels and a lesser but equally significant reduction in total tau. EIF2AK2 has been shown to be activated in AD [185] and has been implicated in the propagation of neuronal apoptosis secondary to Aβ toxicity [186]. The results from the Azorsa study suggest important roles for EIF2AK2 in the expression of total and phosphorylated tau proteins in AD and the inhibitory effects of siRNAs on tau pathology in AD [184].

We and others have shown that siRNAs targeting BACE1 significantly reduce Aβ levels. Employing four siRNAs targeting the catalytic and adjoining regions of BACE1, we found that an 18% reduction in BACE1 mRNA resulted in an 83% decrease in Aβ secreted from APP-transfected SH-SY5Y cells [187]. In 2008, Faghihi and colleagues reported a novel long non-coding antisense BACE1 transcript (BACE1-AS) that consisted of a ~2-kb RNA sequence that was transcribed from the opposite strand of the BACE1 locus. The encoded transcript formed an RNA duplex with the BACE1 mRNA, increasing its stability [188]. *BACE1-AS* transcript levels were found to be elevated 2–3-fold in the parietal cortex and cerebellum of the brains of AD subjects. Interestingly, we reported increased BACE1 protein expression in the AD brain and cerebrospinal fluid [15,16,17] in the absence of changes in *BACE1* mRNA [15]. These findings by Faghihi and colleagues [188] probably explain our results, whereby the stability of *BACE1* mRNA would lead to increased expression of its protein due to a loss in turnover. This in turn would lead to increased production of CTFβ and Aβ and increased senile plaque accumulation [15]. Aβ_42_ accumulation also appears to enhance *BACE1-AS* expression, contributing to Aβ_42_ deposition via positive feedback [188]. Studies by Zhang et al. [189] demonstrated that *BACE1-AS* siRNA transfection into SAMP8 mice significantly enhanced neuronal proliferation, reduced amounts of BACE1, APP, and phosphorylated tau, inhibited Aβ_40_ and Aβ_42_ deposition in the hippocampus, and significantly improved the memory and learning of the mice within 3 weeks of lentiviral infection with *BACE1-AS* siRNA [189].

While a number of studies focusing on the roles and mechanisms of siRNA-induced RNA interference and possible therapeutic efficacy in Alzheimer’s disease have been conducted, more of these studies need to be undertaken, especially in greater detail, so as to identify other nuclear elements, proteins, and pathways that RNA interference may influence to either contribute to or mitigate disease progression.

### 4.3. Roles of Piwi-Interacting RNAs in Alzheimer’s Disease Pathophysiology

Extensive research on the roles of piRNAs in the crucial mechanisms that promote Alzheimer’s disease onset and progression is still ongoing. However, a few studies have described some of their roles in the regulation of Aβ levels in AD and AD-related oxidative stress and apoptosis. The first major study to confirm a role for piRNAs in the human brain in AD was conducted by Roy et al. [190]. They found that 125 different piRNAs were directly involved in the downregulation or silencing of 1923 different mRNAs in AD. The four most important genes affected by the piRNAs were CYCS, LIN7C, KPNA6, and RAB11A, with resultant effects on a number of molecular pathways involved in the disease process [190]. The piRNAs piR-34393 and piR-38240 were noted to be particularly important in AD pathogenesis, as they were observed to reduce the expression of cytochrome C somatic (CYCS) and KPNA6, which codes for Karyopherin α6. The dysregulation of both of these proteins in AD has been established. Perturbations in cytochrome functions may impair mitochondrial ATP generation, promote electron leaks, and consequently produce free radicals within the mitochondria, while Karyopherin plays important roles in the maintenance of cellular homeostasis during states of oxidative stress, mediates nucleocytoplasmic protein transport via importins and exportins, and, via its actions as chaperones, prevents abnormal protein aggregation [191,192]. These findings indicate that piRNAs may promote oxidative stress and the accumulation of insoluble protein aggregates (including Aβ and tau), which contribute significantly to AD onset and progression. In addition, piRNAs have also been shown to be involved in the initiation of a number of single nucleotide polymorphisms (SNP) associated with AD, with Mao et al. [193] identifying 103 piRNAs associated with SNPs in AD.

### 4.4. Roles of Circular RNAs in Alzheimer’s Disease Pathophysiology

As noted earlier, circular RNAs typically function as microRNA sponges in mammalian cells, where they have been implicated in pathogenic processes involved in many human disease conditions, including neurological disorders [194]. Circular RNAs influence AD onset and progression via the roles they have been noted to play in disease pathomechanisms, including Aβ deposition, oxidative stress, and neuroinflammation. Several studies have shown that miR-138 exerts influences on learning and memory by regulating the activities of acyl protein thioesterase 1 (APT1) [195,196]. Hansen et al. [197], in one of the earliest functional analyses of naturally expressed circRNAs, showed that the testis-specific circular RNA produced from the Sry (Sex-determining region Y) gene serves as a sponge for miR-138 and may therefore influence memory and learning behaviours.

The circular RNA ciRS-7, derived from the antisense cerebellar degeneration-related protein 1 (CDR1-AS) gene, is known to act as an endogenous sponge for miR-7 [198]. The circular RNA Cdr1as, via miR-7 and its targets, regulates insulin transcription and secretion in islet cells [198]. MiR-7 has been shown to be significantly elevated in AD brains, influencing Aβ deposition [199]. Subsequently, Zhao and colleagues [200] reported low ciRS-7 levels in the neocortex and hippocampal area CA1 in AD subjects compared to controls. This decrease in ciRS-7 would decrease the ‘sponging’ activity of miR-7, resulting in increased levels and subsequent propagation of the amyloidogenic pathway via increased expression of BACE1 and APP and down regulation of a number of mRNA targets, including Ubiquitin protein ligase A (UBE2A), an autophagic protein important for amyloid peptide proteolysis and clearance via the ubiquitin-26S proteasome pathways [200]. Downregulation or dysfunction of components of the proteasome system has been shown to be associated with Aβ accumulation and senile plaque deposits in AD [201,202]. Interestingly, ciRS-7 may also offer neuroprotective effects in AD as, based on studies by Shi et al. [203], it may increase the rates of APP and BACE1 degradation via a different ubiquitin-proteasome system as it up regulates the expression of ubiquitin carboxyl-terminal hydrolase L1 (UCHL1) mRNA and protein, consequently reducing Aβ generation.

Some circular RNAs have been recently reported to play important regulatory roles in oxidative stress. Huang and colleagues [204] reported increased expression of mmu_circRNA_013636 and reduced expression of mmu_circRNA_012180 in the hippocampus of SAMP8 AD mice. These two circRNAs were predicted to interact and regulate 462 and 631 mRNAs [204]. Subsequent treatment of these mice with panax notoginseng saponins (PNS), the main active compound extracted from the root of panax notoginseng, reversed the expression of these particular circRNAs [204]. Earlier studies by Huang and co-workers demonstrated that treatment of SAMP8 AD mice with PNS prevented oxidative stress injury by increasing gene expression and activity of important components of the enzymatic antioxidant system, including catalase (CAT), superoxide dismutase (SOD), and glutathione peroxidase (GSH-Px) [205]. Reversing the above-mentioned circular RNA profile suggests that circular RNAs play important roles in oxidative stress in AD, and the regulation of circRNAs may hold potential therapeutic benefits. The findings in mice and human tissues are summarised in Table 2.

Circular RNAs have also been shown to exert influences on neuroinflammatory mechanisms in AD. Yang et al. [206] showed that circRNA_0000950, via its actions as a sponge for miR-103, increases the levels of prostaglandin endoperoxide synthase 2 (PTGS2) and the inflammatory cytokines IL-1β and TNF-α, whilst also suppressing neurite outgrowth and promoting neuronal apoptosis in cellular AD models. Studies by Diling and colleagues [207] revealed that circNF1-419, via its interaction with adaptor protein 2 B1 (AP2B1), regulated inflammatory factors TNF-α and NF-κB, with a resultant downregulation of tau, phosphorylated tau, Aβ_1-42_, and APOE, delaying AD onset. Zhang and colleagues [208] showed that berberine-induced upregulation of circHDAC9 (Histone deacetylase 9) reduced Aβ_42_-induced neuroinflammation in human neurons via its actions as a sponge for miR-142-5p, as evidenced by a reduction in the activity of caspase-3 and levels of interleukin-1β (IL-1β), IL-6, and tumour necrosis factor-α (TNF-α).

### 4.5. Roles of Long Non-Coding RNAs in Alzheimer’s Disease Pathophysiology

As discussed earlier, long non-coding RNAs have a predominantly nuclear distribution, and their functions may vary depending on the cell type. With diverse functional mechanisms, including epigenetic regulation, dysregulation of lncRNA function has been linked to the pathophysiological processes involved in cancer, epilepsy, cardiovascular disease, and various genetic and neurodegenerative diseases, including Alzheimer’s disease [209]. Several lncRNAs, including BACE1-AS, MALAT1, 51A, 17A, NDM29, BC200, NAT-Rad18, and BDNF-AS are known to regulate APP processing, tau phosphorylation, synaptic plasticity, and neuroinflammation and have been shown to play varying roles in AD pathogenesis [210].

#### 4.5.1. Roles of LncRNAs in Aβ Production and Clearance

BACE1-AS is a lncRNA that is transcribed from the sense strand of the BACE1 gene and functions as a competing endogenous RNA (ceRNA) [188]. The lncRNA shares miRNA-response elements, including miR-29, miR-107, miR-124, miR-485, and miR-761 [188]. BACE1-AS has been shown to upregulate BACE1 expression in AD subjects [211], and BACE1-AS itself has been shown to be highly expressed in the blood and brain of AD patients and in AD animal models, thus facilitating AD progression via its effects on BACE1 activity. Studies have also shown that knockdown of BACE1-AS decreases BACE1 and Aβ levels, inhibits tau phosphorylation in the hippocampus, and improves learning performance and memory in AD mice [189,212].

Another lncRNA that has been found to be highly expressed in the AD brain is BC200, also referred to as brain cytoplasmic RNA 1 (BCYRN1) [213]. BC200 promotes AD progression by modulating BACE1 expression, resulting in elevated levels of Aβ production [213]. Inhibition of this lncRNA in a cell culture model Aβ_1–42_ suppressed BACE1 expression, increased neuronal viability, and reduced neuronal loss [214].

The nuclear enriched abundant transcript 1 (NEAT1) lncRNA is also known to play a role in AD pathogenesis, with its levels shown to be elevated in AD [215]. Zhao and colleagues [216] have shown that NEAT1 inhibits the actions of miRNA-124, a microRNA that inhibits BACE1 expression (and Aβ production) by binding to its 3′-UTR. Thus, NEAT1 plays a vital role in AD pathogenesis, and its knockdown may produce protective effects in AD.

Sortilin-related receptor 1 (SORL1) functions as a sorting receptor for APP and plays a critical role in preventing AD progression, as a reduction in its expression promotes Aβ formation and aggregation in the brains of AD subjects [217]. Downregulation of SORL1 redirects APP to the β-secretase cleavage pathway, promoting Aβ formation [218]. LncRNA 51A is an antisense configuration of the first intron of the *SORL1* gene that inhibits SORL1 expression, and as such, this lncRNA may promote Aβ generation in AD [219].

Low-density lipoprotein receptor-related protein 1 (LRP1) plays a critical role in CNS function. In addition to being important for cellular cholesterol transport, LRP1 also plays roles in the endocytosis of ligands and transcytosis across the blood-brain barrier (BBB) [220]. BBB integrity [221] is important for Aβ clearance, with its expression noted to be lower in AD subjects [222]. Yamanaka and colleagues [223] showed that LRP1-AS, a conserved antisense lncRNA transcribed from the *LRP1* gene locus, decreases LRP1 expression in the brains of AD subjects, possibly via epigenetic mechanisms, with consequent impairment of Aβ clearance, leading to amyloid plaque aggregation.

#### 4.5.2. Roles of LncRNA in Tau Hyperphosphorylation and Neuronal Loss

In addition to its influences on Aβ production, NEAT1 also appears to play a role in tau hyperphosphorylation in AD. Knockdown of NEAT1 has been shown to elevate expression of p-tau via the frizzled class receptor 3 (FZD3)/CSK3β/p-tau pathway, with metformin inducing a reversal of these effects as it increases NEAT1 expression [224,225].

Linc00507, a member of the long intervening non-coding RNA (lincRNA) family, a subclass of long non-coding RNAs, is expressed almost exclusively in the cortex in primates and humans [226]. It has been shown to be upregulated in both the cortex and hippocampus of APP/PS1 mice and, acting via the p25/p35/GSK3β pathway, trigger extensive tau phosphorylation and aggregation [226]. It may also produce this effect by upregulating MAPT and tau tubulin kinase via its actions on miR-181C-5P [227].

LncRNA EBF3-AS, a 2-exon RNA consisting of 842 nt, is transcribed from the opposite strand of the protein-coding gene early B cell factor 3 (*EBF3*) located on chromosome 10 [228]. EBF3 is a DNA-binding transcription factor that inhibits cell proliferation and induces cell cycle arrest, growth suppression, and apoptosis [229]. Magistri et al. [228] showed that EBF3-AS was upregulated in the brains of late-onset Alzheimer’s disease (LOAD) subjects, while Gu and colleagues [230] later corroborated this finding in the hippocampus of APP/PS1 AD mice and showed that it regulates EBF3 expression and, importantly, promotes Aβ-induced neuronal apoptosis in AD subjects.

Another lncRNA that has been associated with neuronal loss in AD is NAT-Rad18. This lncRNA is known to play important roles in DNA repair [231,232] and is expressed in the hippocampus, cortex, striatum, cerebellum, brain stem, spinal cord, and olfactory bulb. Harvey and colleagues [233] showed that NAT-Rad18 upregulation increases the sensitivity of cells to potentially genotoxic agents, contributing to Aβ-induced neuronal apoptosis.

#### 4.5.3. Roles of LncRNAs in Neuroinflammation and Oxidative Stress

Maternally expressed gene 3 (MEG3), located on chromosome 14q32.3 in humans, encodes for a lncRNA of ~1700 bp and has been found to play important roles in the mediation of neuroinflammation in Alzheimer’s disease via its effects on microglia and astrocytes [234], amongst other functions. MEG3 inactivates the functionally pivotal PI3/Akt signalling pathway in astrocytes via inhibition of critical protein components, which leads to improved spatial memory in AD mice [235]. MEG3 may also act to promote microglia activation in AD via the inhibition of miR-7a-5p [234].

Metastasis-associated lung adenocarcinoma transcript 1 (MALAT1), also known as noncoding nuclear-enriched abundant transcript 2 (NEAT2) is a 6.7–7 kb lncRNA transcribed from a locus on Chromosome 11q13 [236]. Studies have demonstrated the active involvement of MALAT1 in numerous physiological processes, including the splicing of various mRNA transcripts, epigenetic modification, and synapse formation [237]. An increase in MALAT1 expression following damage induced by hypoxia/ischemia in mice has been shown to decrease the susceptibility of the brain to injury by promoting angiogenesis, inhibiting apoptosis, and inflammation, and regulating autophagic changes in the brain [238]. Other studies have revealed that in addition to reducing neuronal apoptosis and promoting functional neuronal repair and regeneration, MALAT1 upregulation also resulted in the downregulation of IL-6 and TNF-alpha levels and the elevation of IL-10 levels, typifying the potent anti-inflammatory actions of this lncRNA [239]. In addition, the study also showed that MALAT1-induced inhibition of miR-125b was associated with a significant reduction in the release of inflammatory cytokines.

A novel lncRNA, ANRIL (lnc-antisense non-coding RNA in the INK4 locus), has been shown to influence important inflammatory processes in various diseases [240,241]. Studies in nerve growth factor (NGF)-treated PC12 cells that were challenged with Aβ_1–42_ showed that knockdown of ANRIL significantly decreased levels of apoptosis and increased neurite outgrowth. These effects were facilitated by significant decreases in the inflammatory cytokines tumour necrosis factor-α (TNF-α), IL-1β, IL-6, and IL-17 [242]. In a study investigating the role of ANRIL in a primary neuron cellular model of hypoxia, Li and colleagues [243] discovered that while hypoxia caused significant induction of ANRIL and cell death, inhibition of ANRIL exacerbated cell death, possibly suggesting a protective role played by this lncRNA in vitro. The implications of these findings need further exploration.

The lncRNA named 17A, discovered in 2007, is a pol-III-dependent ncRNA that harbours the potential to regulate pol-II-transcribed protein-coding genes. This lncRNA maps to intron 3 of the *GPR51* gene that codes for the GABA B2 receptor (GABAB R2). Expression of 17A has been found to tightly regulate the splicing of GPR51, leading to the generation of four variants of GABAB R2, favouring the non-functional variant B [244]. Since the functioning of GABAB R2 requires a heterodimeric complex forming between GABA B1 and GABA B2, the predominant generation of variant B2 in 17A ncRNA-expressing cells significantly impairs GABAB signalling [244]. Levels of 17A have also been shown to be upregulated in the AD brain, and it exerts influences on the regulation of neuroinflammation and clinical phenotype [244,245], typically via its effects on GABARs. The GABA-B receptors are also involved in the regulation of microglial function and general modulation of neuroinflammation as they are known to inhibit the release of pro-inflammatory cytokines. The 17A cells have been shown to downregulate the expression of GABA-B receptors and deactivate the GABA-B signaling cascade in AD mouse models [244,246], implying their potential relevance in the neuroinflammatory processes involved in AD pathophysiology, which must be further explored.

Another lncRNA that has been implicated in neuroinflammation in AD is MAGI2-AS3, transcribed from the antisense strand of the membrane-associated guanylate ginase, WW, and PDZ domain containing 2 (MAGI2) gene located at chromosome 7q21.11 (genecards.org). It was initially known to play varying roles in processes that promote or inhibit the progression of various neoplastic diseases [247,248] but has recently been shown to play roles in Aβ-mediated neuroinflammation in AD, with studies revealing that the upregulation of MAGI2-AS3 promotes Aβ deposition, neuroinflammation, and neuronal loss [249].

The lncRNA SOX21-AS1, another antisense transcript that targets FZD3/5 and regulates Wnt signalling has been shown to play important roles in oxidative stress-induced neuronal injury and loss in AD mouse models. Zhang and colleagues showed that levels of SOX21-AS1 are elevated in the hippocampus of AD mice and that its downregulation may result in increased FZD3/5 expression and activation of Wnt signalling, which offers substantial protection against oxidative stress, reduces neuronal loss, and ultimately improves learning abilities and memory in AD [250].

The lncRNA activation by transforming growth factor-β (lncRNA-ATB) is also involved in oxidative stress in AD, as elevated levels have been shown to be associated with Aβ-induced oxidative stress and neurotoxicity. Wang and co-workers showed that suppression of this lncRNA significantly reduces oxidative damage in a cellular model of AD, possibly as a result of its regulatory effects on miR-200/ZNF217 [251].

Table 3 provides a summary of some dysregulated lncRNAs found in AD tissues.

## 5. Prospects of Non-Coding RNAs as Potential Therapeutic Targets and Biomarkers for Alzheimer’s Disease

The discovery of the roles that ncRNAs play in various pathogenic processes in Alzheimer’s disease provides a new perspective for further understanding the disease process and developing new therapeutic options for the disease. Some of the numerous mechanisms by which non-coding RNAs influence the pathophysiological processes involved in Alzheimer’s disease have been extensively discussed. These influences imply that non-coding RNAs can be employed, targeted for therapeutic benefits, or used as biomarkers for Alzheimer’s disease.

Non-coding RNA-based therapies have already been developed for a wide range of disease conditions [252] and studies are ongoing to identify viable non-coding RNA therapeutic targets for Alzheimer’s disease. MicroRNAs and long non-coding RNAs are two families of ncRNAs that have been best studied for their potential as therapeutic targets for Alzheimer’s disease. Many of the studies that have been highlighted in this review suggest that ncRNAs can be manipulated, depending on their intrinsic contributions to AD pathophysiology, in order to slow down disease progression and produce beneficial clinical effects. The expression profile of non-coding RNAs in the brains of AD patients with respect to different pathological processes reflects their potential as therapeutic targets. The levels of a number of microRNAs are altered during specific Braak stages in AD patients, and changes in the expression of certain miRNAs are observed throughout the disease process, from early stages characterised clinically by mild cognitive impairment to the later, more clinically severe stages. As described in previous sections of this review, the dysregulation of miRNAs in the brains of AD patients and animal models affects the pathological progression of AD by regulating many target genes and signalling pathways and may be manipulated for therapeutic benefits in a wide variety of neurodegenerative conditions [253]. MicroRNA mimetic activity represents a new approach to miRNA therapeutics. They are exogenously synthesised double-stranded RNA molecules that are subsequently processed and modified in vivo into functional microRNAs [254,255]. Other miRNA mimics may be designed to inhibit the functions of endogenous miRNAs, and they are typically designed based on the complementary sequence of the target miRNA.

Long non-coding RNAs also exert significant influences on various pathophysiological processes in Alzheimer’s disease, and therapeutic measures to target them are also being developed. Oligonucleotide compounds, antisense oligonucleotides, and small interfering RNAs are being investigated for their ability to target and knockout specific lncRNAs and harness therapeutic effects [256,257].

Oligonucleotides, antibodies, and other small molecules are also being explored for their ability to precisely target ncRNAs for therapeutic benefits [258]. These molecules can gain entry into cells and specifically target RNAs that are ordinarily not easily accessible to other types of therapeutic compounds or substances that rely on cell receptor activation [259,260]. The high specificity of oligonucleotides in binding RNA targets will also result in a significantly minimal side effect profile.

An important challenge that ncRNA-based therapy for Alzheimer’s disease faces is the blood-brain barrier, and strategies such as lipid or polymer nanoparticle delivery systems [261,262], focused ultrasound [263], and adeno-associated virus vectors [264] are being investigated to circumvent this challenge.

Non-coding RNAs circulating in the serum or CSF are also potentially useful as biomarkers for the early detection of Alzheimer’s disease based on the changes in their expression during the disease process [265]. A number of studies have identified the potential usefulness of specific miRNAs and piRNAs as diagnostic or prognostic markers for Alzheimer’s disease. There are advantages to using circulating miRNAs as biomarkers apart from their close association with diseases: the ease of use of miRNA detection technology, the resistance of miRNAs to RNase digestion and tolerance of a wide range of pH conditions, and their stability at room temperature [147]. These advantages suggest the possibility of miRNAs and possibly other ncRNAs as ideal biomarkers for AD. Early diagnosis may bring about interventions that significantly delay or even prevent Alzheimer’s disease onset, and more studies to identify rapid and non-invasive biomarkers must be embarked upon in order to improve early diagnosis.

A major phenomenon that should also be explored in future studies is the existence of different types of RNA fragments within protein deposits in AD. Shmookler Reis and colleagues [266] demonstrated a significant, non-random presence of RNA within pathological protein aggregates in AD states, and the significance of this finding needs to be studied in detail, especially as regards its cause and effects on AD pathophysiology.

## 6. Conclusions

Non-coding RNAs perform critical regulatory functions in a wide range of cell types. Via epigenetic mechanisms and by regulating the expression of a number of genes, ncRNAs exert influence over a potentially wide range of cellular processes that depend on the expression and function of a large number of proteins. The wide-ranging effects of ncRNAs on normal cell function imply that their actions and dysregulation could have important roles in the onset and progression of many human diseases. Non-coding RNAs have been found to play important roles in the pathophysiology of Alzheimer’s disease, with recent studies in cellular and animal models as well as human brain tissue, serum, and CSF studies shedding light on some of these roles. MicroRNAs, circular RNAs, piwi-interacting RNAs, small interfering RNAs, and long non-coding RNAs have all been implicated in amyloid plaque formation and accumulation, tau hyperphosphorylation, neuroinflammation, oxidative stress, autophagy, and many other important pathophysiological processes in Alzheimer’s disease onset and progression. Many of these studies also reveal that some non-coding RNAs can be manipulated in AD, with potential therapeutic changes to the pathophysiological process. In addition, due to changes in the expression pattern of many non-coding RNAs at different stages of the disease, non-coding RNAs may also be useful as biomarkers for Alzheimer’s disease, and a number of studies have generated results that support this idea. Using non-coding RNAs as biomarkers for AD may significantly improve early detection and ultimately result in better clinical outcomes resulting from earlier intervention.

Although significant strides have been made in the study of the roles that non-coding RNAs play in AD pathogenesis, much still remains unknown with regard to the exact mechanistic nature of their pathophysiological roles and their potential as therapeutic options and biomarkers. More detailed studies on the properties of many non-coding RNAs, the exact roles that they play in AD progression, and how they play these roles will lead to the discovery of new biomarkers and viable therapeutic strategies for the disease, which will improve prognosis and outcomes for patients.

## Figures and Tables

**Table 1:** Examples of dysregulated miRNAs in AD.

Non-Coding RNA	Expression	Source/Specimen	References
miR-16; miR-29a/b-1 and c; miR-186; miR-195	Decreased	AD brain; Tg mouse brain	[5,137,138,139,140]
miR-101; miR-101a-3p; miR-346; miR-384	Decreased	AD brain and Tg mouse brain	[146,147,148]
miR-146a	Increased	AD brain	[154]
miR-512	Decreased	AD brain	[156]
miR-206	Increased	AD serum	[161]
miR-133b	Decreased	AD brain	[164]
miR-222	Decreased	AD serum	[166]
miR-132; miR-212	Increased	Lymphoblastoid cells from AD patients	[170]

**Table 2:** Summary of circular RNA activity in the context of AD.

Non-Coding RNA	Expression	Source/Specimen	Reference
ciRS-7	Increased	AD brain, blood	[199]
ciRS-7	Decreased	AD cortex and hippocampus (CA1)	[200]
mmu_circRNA_013636	Increased	SAMP AD mice	[204]
mmu_circRNA_012180	Decreased	SAMP AD mice	[204]

**Table 3:** Examples of dysregulated lncRNAs in AD tissues.

Non-Coding RNA	Expression	Source/Specimen	References
BACE1-AS	Increased	AD brain, blood	[161]
BC200/BCYRN1	Increased	AD brain	[213]
NEAT1	Increased	AD brain	[215]
LRP1-AS	Increased	AD brain	[223]
Linc00507	Increased	APP/PS1 mouse brain	[226]
EBF3-AS	Increased	AD brain, APP/PS1 mouse brain	[228,230]
17A	Increased	AD brain	[244,245]
SOX21-AS1	Increased	Hippocampus of AD mice	[250]

## Data Availability

All data are presented in the article.
